# Transactions of New York Odontological Society

**Published:** 1889-05

**Authors:** 


TRANSACTIONS OF NEW YORK ODONTOLOGICAL SOCIETY.—Regular Meeting, 1888. S. S. White Dental Manufacturing Company, Philadelphia. The volume contains an interesting and valuable report of the proceedings of this prominent Society and contains useful information which is not published in any of the current literature of the profession. Some of the articles are illustrated, and the volume is in its usual attractive style and binding.

